# MicroRNAs in Plant Genetic Regulation of Drought Tolerance and Their Function in Enhancing Stress Adaptation

**DOI:** 10.3390/plants14030410

**Published:** 2025-01-30

**Authors:** Yryszhan Zhakypbek, Ayaz M. Belkozhayev, Aygul Kerimkulova, Bekzhan D. Kossalbayev, Toktar Murat, Serik Tursbekov, Gaukhar Turysbekova, Alnura Tursunova, Kuanysh T. Tastambek, Suleyman I. Allakhverdiev

**Affiliations:** 1 Department of Surveying and Geodesy, Mining and Metallurgical Institute Named After O.A. Baikonurov, Satbayev University, Almaty 050043, Kazakhstan; murat-toktar@mail.ru (T.M.); s.tursbekov@satbayev.university (S.T.); 2 Department of Chemical and Biochemical Engineering, Geology and Oil-Gas Business Institute Named After K. Turyssov, Satbayev University, Almaty 050043, Kazakhstan; kossalbayev.bekzhan@gmail.com; 3 Department of Biotechnology, Al-Farabi Kazakh National University, Almaty 050040, Kazakhstan; 4 Ecology Research Institute, Khoja Akhmet Yassawi International Kazakh Turkish University, Turkistan 161200, Kazakhstan; tastambeku@gmail.com; 5 Sustainability of Ecology and Bioresources, Al-Farabi Kazakh National University, Al-Farabi 71, Almaty 050038, Kazakhstan; 6 Department of Agronomy and Forestry, Faculty of Agrotechnology, Kozybayev University, Petropavlovsk 150000, Kazakhstan; 7 Department of Soil Ecology, Kazakh Research Institute of Soil Science and Agrochemistry, Named After U.U. Uspanov, Al-Farabi Ave. 75, Almaty 050060, Kazakhstan; 8 Department of Metallurgy and Mineral Processing, Satbayev University, Almaty 050000, Kazakhstan; ipgauhartas@gmail.com; 9 Kazakh Research Institute of Plant Protection and Quarantine Named After Zhazken Zhiembayev, Almaty 050070, Kazakhstan; alnura_89.12.12@mail.ru; 10 Department of Plant Physiology, Faculty of Biology, M.V. Lomonosov Moscow State University, Leninskie Gory 1-12, 119991 Moscow, Russia; suleyman.allakhverdiev@gmail.com; 11 Controlled Photobiosynthesis Laboratory, K.A. Timiryazev Institute of Plant Physiology RAS, Botanicheskaya Street 35, 127276 Moscow, Russia; 12 Faculty of Engineering and Natural Sciences, Bahcesehir University, Istanbul 34353, Turkey

**Keywords:** microRNA, mRNA, abiotic stress, drought stress, gene expression

## Abstract

Adverse environmental conditions, including drought stress, pose a significant threat to plant survival and agricultural productivity, necessitating innovative and efficient approaches to enhance their resilience. MicroRNAs (miRNAs) are recognized as key elements in regulating plant adaptation to drought stress, with a notable ability to modulate various physiological and molecular mechanisms. This review provides an in-depth analysis of the role of miRNAs in drought response mechanisms, including abscisic acid (ABA) signaling, reactive oxygen species (ROS) detoxification, and the optimization of root system architecture. Additionally, it examines the effectiveness of bioinformatics tools, such as those employed in in silico analyses, for studying miRNA-mRNA interactions, as well as the potential for their integration with experimental methods. Advanced methods such as microarray analysis, high-throughput sequencing (HTS), and RACE-PCR are discussed for their contributions to miRNA target identification and validation. Moreover, new data and perspectives are presented on the role of miRNAs in plant responses to abiotic stresses, particularly drought adaptation. This review aims to deepen the understanding of genetic regulatory mechanisms in plants and to establish a robust scientific foundation for the development of drought-tolerant crop varieties.

## 1. Introduction

Due to global climate change and increasing anthropogenic impacts, drought has become a pressing issue worldwide [1,2]. Terrestrial plants are among the first to suffer from drought’s effects. Over the course of evolution, plant species in arid climatic zones have developed various morphological, physiological, biochemical, and genetic adaptations to counteract stress factors like drought [3,4,5]. Despite extensive research on the molecular and genetic mechanisms underlying plant stress adaptation, our understanding of plants’ responses to drought stress remains incomplete [6,7]. Recent studies, however, have identified that one mechanism through which plants respond to drought involves the regulation of gene expression by small non-coding RNAs, specifically microRNAs (miRNAs) [8,9].

MicroRNAs are small, non-coding RNA molecules of 21–25 nucleotides that serve as key regulators of gene expression in plants [10,11,12]. They enable molecular adaptation during developmental processes and in response to environmental stimulus by controlling gene expression at spatial and temporal levels [13,14]. MicroRNAs play an essential role in regulating numerous physiological processes during plant development. For example, miR166 contributes to vascular tissue formation, whereas miR156 is key in managing shifts between developmental phases [15,16]. Furthermore, miRNAs display distinct spatial and temporal expression patterns, with miR167 predominantly active in roots and miR172 more prevalent in leaves, thereby supporting specific organ functions and influencing flowering time [17,18]. These insights emphasize the intricate regulatory mechanisms of miRNAs across various stages of growth and within different plant tissues. Additionally, miRNAs regulate crucial biological processes such as cellular differentiation, proliferation, and cell viability [11,19]. Recent studies show that drought conditions alter miRNA expression levels across various plant species [20,21], leading to changes in the expression of their target genes in response to drought stress. For example, increased expression of drought-responsive miRNAs has improved drought tolerance in transgenic plants [22,23,24].

An in-depth investigation of the molecular–genetic mechanisms underlying plant responses to drought stress is essential for enhancing agricultural productivity and developing climate-resilient crop varieties [25,26]. This review summarizes recent molecular–genetic studies on miRNAs involved in the regulation of drought-responsive genes, with a focus on the regulatory networks associated with drought-responsive miRNAs.

## 2. Mechanism of miRNA in the Genetic Regulation of Drought Stress Tolerance in Plants

### 2.1. MicroRNA Biogenesis and Processing Pathway in Plants

**Figure 1:**
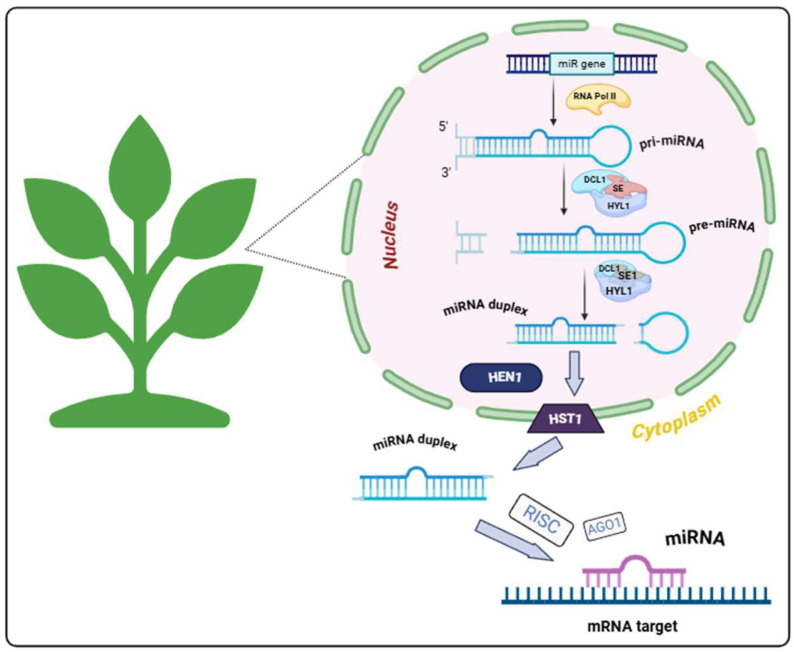
Biogenesis and processing pathway of miRNAs in plants. The miRNA gene is transcribed by RNA polymerase II into a pri-miRNA with a stem-loop structure. The pri-miRNA is processed by the DCL1 enzyme, along with auxiliary proteins such as SE and HYL1, resulting in the formation of a pre-miRNA molecule, which subsequently develops into a miRNA duplex. Then, this duplex is stabilized by methylation from the HEN1 enzyme, after which it is exported from the nucleus to the cytoplasm via the HASTY (HST1) protein for further integration into the RISC. In the cytoplasm, the mature miRNA strand is loaded into the RISC, where it guides the AGO1 protein to bind complementary target mRNA, leading to mRNA cleavage or translational repression.

In plants, the biogenesis of miRNAs involves several key stages that are essential for their regulatory functions [27]. MicroRNAs are produced through the transcription of miRNA genes, which can reside within the introns of protein-coding genes or be transcribed as independent units [24]. The process begins with the transcription of primary miRNA (pri-miRNA) genes by RNA polymerase II, resulting in long, single-stranded RNA molecules that possess one or more hairpin-like structures [28,29]. Within the nucleus, pri-miRNAs are recognized and processed by a protein complex known as the microprocessor, primarily composed of Dicer-like 1 (DCL1) enzyme and cofactors, such as Serrate (SE) and Hyponastic leaves 1 (HYL1) [30,31]. The DCL1 enzyme processes pri-miRNA, cutting it into a hairpin-structured precursor miRNA (pre-miRNA), which is then stabilized by methylation from the enzyme HEN1 and transported to the cytoplasm with the help of the HASTY (HST1) protein. In the cytoplasm, the pre-miRNA undergoes further processing by the DCL, resulting in the formation of a duplex that matures into single-stranded miRNA [32]. The mature miRNA is then integrated into the RNA-induced silencing complex (RISC), where it interacts with its complementary target mRNA. This interaction activates the catalytic component of the RISC, the AGO1 protein, leading to the repression or cleavage of the target mRNA’s translation (Figure 1) [33,34]. This biogenesis process in plants underscores the crucial role of miRNAs in regulating gene expression in response to environmental factors, including abiotic stresses such as drought [35].

The biogenesis of miRNAs in plants is a multi-step, highly coordinated process that plays a crucial role in post-transcriptional regulation of gene expression [36]. From the transcription of pri-miRNAs to their final integration into the RISC, each stage ensures precise and efficient control of gene expression [37,38]. This process is essential for maintaining cellular homeostasis and enabling plants to adapt effectively to various environmental stress factors, including drought. A deeper understanding of the intricacies of miRNA biogenesis not only enhances our knowledge of plant molecular biology but also opens new avenues for improving crop resilience through genetic and biotechnological approaches [39,40]. This is particularly important for increasing the adaptability of plants to changing environmental conditions, especially in the face of drought and other abiotic stresses [41].

### 2.2. Signaling Pathways Associated with Stress Tolerance Regulated by miRNAs

**Figure 2:**
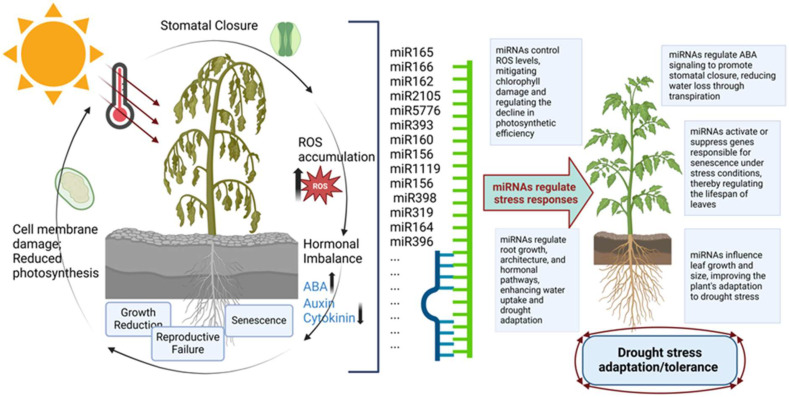
Role of miRNAs in drought stress adaptation and tolerance. Drought stress in plants leads to stomatal closure, reducing water loss, while the accumulation of ROS at the cellular level induces oxidative damage, causing membrane disruption, hormonal imbalance, growth reduction, premature senescence, and reproductive failure. MicroRNAs regulate the interplay of phytohormones such as ABA, auxin, and cytokinin, optimizing plant adaptation to drought conditions and enhancing drought stress adaptation and tolerance.

MicroRNAs play a critical role in plant adaptation to stress conditions by regulating several key signaling pathways [42,43]. Among these, hormonal signaling pathways, antioxidant-based pathways, calcium-dependent pathways, and mechanisms involving natural antisense transcripts are of particular importance (Table 1) [44]. As shown in Figure 2, miRNAs effectively regulate plant stress responses by modulating processes such as stomatal closure, reactive oxygen species (ROS) detoxification, hormonal balance, and the restructuring of root and leaf architecture. These mechanisms optimize plant adaptation to drought conditions and enhance tolerance by stabilizing cellular homeostasis, improving water use efficiency, and reducing oxidative damage (Figure 2).

#### 2.2.1. MicroRNAs in Hormonal Signaling Pathways

In hormonal signaling pathways, abscisic acid (ABA) functions as a pivotal phytohormone in response to drought and salinity stress [45]. It plays a crucial role in mediating stress signals, regulating stomatal closure, and modulating gene expression. Additionally, ABA is synthesized in dehydrating roots during drought conditions, where it inhibits lateral root growth, contributing to the plant’s adaptive mechanisms under stress [46,47]. MicroRNAs play a critical role in regulating hormonal signaling pathways, which are essential for plant adaptation to stress conditions. For instance, miR159 plays a vital role in enhancing drought tolerance, particularly by targeting MYB transcription factors involved in stress and ABA signaling. In Populus and Arabidopsis thaliana (A. thaliana), overexpression of miR159 improves water use efficiency, promotes stomatal closure, enhances ROS scavenging, and reduces cellular damage, thereby increasing drought resilience. Conversely, in rice, the downregulation of miR159 may activate MYB factors, suggesting a species-specific mechanism for stress adaptation. These findings highlight miR159’s potential in developing drought-tolerant crops [48,49,50,51]. Furthermore, Yan et al. [52] demonstrated that the miR165/166 regulatory module plays a critical role in maintaining ABA homeostasis and response in A. thaliana. Their study revealed that miR165/166 modulates the expression of key components in ABA signaling and metabolism, specifically ABA INSENSITIVE 4 (ABI4) and β-GLUCOSIDASE1 (BG1). Disruption of miR165/166 function led to altered ABA sensitivity and stress responses, highlighting its essential role in maintaining ABA balance under stress conditions.

Li et al. [53] investigated the role of miR162 in modulating stomatal conductance in response to low-night-temperature stress via the ABA signaling pathway in tomato. Their findings demonstrated that miR162 regulates the expression of DCL1, thereby influencing the biogenesis of other ABA-responsive miRNAs, which in turn modulate stomatal dynamics and enhance stress adaptation. This study highlights the critical function of post-transcriptional modulators as systemic responders within the ABA signaling cascade and provides new insights into post-transcriptional regulation as a mechanism for improving abiotic stress resilience in plants. Recent studies have investigated the function of miR2105 in rice plants under drought conditions. The findings indicate that the downregulation of miR2105 leads to increased expression of the transcription factor OsbZIP86, which activates the NCED3 gene involved in ABA biosynthesis. This activation results in elevated ABA levels, enhancing drought tolerance without compromising yield. Conversely, overexpression of miR2105 renders plants more susceptible to drought, highlighting its critical regulatory role in ABA-mediated drought responses [54,55]. In a 2024 study, researchers conducted an integrated transcriptome and miRNA analysis to investigate the molecular mechanisms underlying bud dormancy in apricot trees. The analysis identified miR5776-x as a key regulator, targeting the PaNCED2 gene involved in ABA biosynthesis. The interaction between miR5776-x and PaNCED2 suggests a critical role in maintaining dormancy through ABA signaling pathways, providing valuable insights into the molecular regulation of dormancy [56]. Zhang et al. [57] explored the roles of various miRNAs in abiotic stress responses, including salinity. They identified that miR393 regulates salinity stress through RACK1A-mediated ABA signaling pathways in Arabidopsis seedlings under high-salt conditions. Similarly, miR393 plays a significant role in drought stress by targeting key components of the auxin signaling pathway, TIR1/AFB2, thereby influencing root growth and architecture under water-deficient conditions. Acting as a negative regulator of ABA, RACK1A mediates the interaction between miR393 and ABA signaling in response to both salinity and drought stress, highlighting the complexity of miRNA-regulated hormonal crosstalk in plant adaptation to abiotic stress [58,59].

Auxin plays a critical role as a key hormone and signaling molecule in regulating various aspects of plant growth and development, including root and leaf architecture, organ patterning, and root development [60], while several miRNA families manage auxin signaling under drought conditions to ensure plant adaptation [61]. A study by Marzi et al. [62] examined the role of transcriptional regulation in auxin-mediated responses to abiotic stresses. The review highlighted that miR160 targets and inhibits Auxin Response Factor 10 (ARF10), a positive regulator of shoot apical meristem (SAM) formation. This interaction underscores the critical role of miR160 in modulating auxin signaling pathways, particularly under stress conditions. The miR160 family plays a critical role in plant growth, development, and stress responses by regulating auxin and cytokinin signaling pathways. The miR160 targets ARFs, including ARF10, ARF16, and ARF17, which are key regulators of auxin signaling. This regulation is essential for balancing the interaction between auxin and cytokinin, influencing processes such as root architecture optimization, nodule development, and tissue regeneration [63,64]. A recent study investigated the effects of miR156ab on plant growth and drought tolerance. The study revealed that overexpression of miR156ab in apple and Arabidopsis plants promoted significant growth, with transgenic lines developing longer roots, taller shoots, and larger leaves under normal conditions. Gene expression analysis showed an upregulation of genes involved in auxin biosynthesis (MdYUCCAs), transport (MdPINs), and signaling (MdLBD18), leading to increased auxin levels and enhanced plant growth [65]. Moreover, in Medicago truncatula (M. truncatula), miR156 enhances drought resilience by increasing root biomass and promoting nodule formation. Additionally, in Triticum dicoccoides and Oryza sativa, miR156 optimizes leaf and root structures, reducing water loss and improving water uptake, thereby strengthening drought adaptation. The role of miR156 in stress adaptation positions it as a promising tool for developing stress-tolerant agricultural crops [66,67,68,69].

#### 2.2.2. MicroRNAs in Antioxidant-Based Pathways

Drought stress increases the accumulation of ROS in cellular compartments such as chloroplasts, peroxisomes, and mitochondria, posing a threat to plant cells [70]. Plants have developed defense systems to regulate ROS levels, including antioxidant enzymes such as superoxide dismutase, catalase, peroxidase, ascorbate peroxidase, and glutathione reductase [71,72,73]. Additionally, miRNAs play a crucial role in maintaining ROS homeostasis by regulating antioxidant enzymes and interacting with hormonal signaling pathways, enhancing plant resilience to stress and enabling adaptation to challenging environmental conditions [74,75]. Recent studies have highlighted the critical role of miRNAs in regulating antioxidant pathways, thereby enhancing plant drought tolerance. These small non-coding RNAs modulate gene expression, reducing oxidative stress and enabling plants to adapt to water-deficient conditions [76]. For instance, under drought conditions, the critical role of miR1119 in regulating oxidative stress has been highlighted. miR1119 induces the expression of genes encoding antioxidant enzymes such as superoxide dismutase and catalase, which are essential for scavenging ROS. This induction enhances the plant’s antioxidant defense system, thereby significantly contributing to improved drought tolerance [77]. A study on tea plants (*Camellia sinensis*) identified members of the miR156 family as key players in drought response. Specifically, csn-miR156f-2-5p was found to target the SQUAMOSA promoter-binding protein-like 14 (CsSPL14), a gene involved in stress responses. The upregulation of csn-miR156f-2-5p under drought stress led to increased ROS accumulation and reduced chlorophyll content, highlighting its role in regulating antioxidant defenses and maintaining photosynthetic efficiency during water deficit conditions [78]. miR398 plays a critical role in regulating antioxidant pathways during drought stress by targeting Cu/Zn superoxide dismutases (CSD1, CSD2) and cytochrome c oxidase (COX5b). Its downregulation enhances CSD activity, improving ROS detoxification and boosting drought tolerance [79].

#### 2.2.3. MicroRNAs in Calcium Signaling and Natural Antisense Transcript-Based Pathways

Calcium (Ca^2+^) serves as a crucial secondary messenger that modulates the complex network of signaling pathways enabling plants to respond to abiotic stresses, particularly drought, and plays a key role in regulating various physiological processes [80]. MicroRNAs act as essential regulators of these signaling pathways, playing a significant role in plant adaptation to water deficiency [81]. Studies have identified miR319 as a key regulator in drought stress adaptation. miR319 targets genes encoding TCP transcription factors, which are involved in various developmental processes and stress responses. Under drought conditions, miR319 modulates TCP transcription factors, influencing calcium-dependent signaling pathways, thereby regulating stomatal behavior and enhancing drought tolerance in plants [82,83]. miR164 regulates NAM, ATAF1/2, and CUC2 (NAC) transcription factors, which play a crucial role in plant development and stress responses. Under drought conditions, miR164-mediated modulation of NACs influences calcium signaling pathways, optimizing root architecture and improving water uptake efficiency [84]. miR396 targets growth-regulating factors (GRFs) involved in cell proliferation and development. Under drought stress, miR396 regulates GRF expression, influencing calcium signaling, modulating leaf growth, and enhancing plant adaptation to stress conditions [77]. These miRNAs highlight the complex regulatory mechanisms plants employ to adapt to drought stress through calcium-dependent pathways. Precise modulation of calcium signaling ensures optimal plant growth and survival under adverse conditions, while understanding their roles provides valuable insights into the molecular basis of drought tolerance and potential targets for developing stress-resilient crops.

Natural antisense transcripts (NATs) are RNA molecules transcribed from the opposite strand of protein-coding or non-coding genes, regulating gene expression through mechanisms such as transcriptional interference, RNA duplex formation, chromatin modification, and miRNA binding [85]. MicroRNAs interact with NATs to precisely coordinate key physiological processes, including stress responses, plant development, and adaptation to drought conditions [86]. For instance, in A. thaliana, miRNA398 and its cis-NATs (such as NAT398b and NAT398c) form a regulatory loop. These NATs suppress pri-miRNA processing, reducing mature miR398 levels and modulating stress responses. Under stress conditions like drought, this regulatory interaction can significantly impact the plant’s ability to manage ROS and maintain cellular homeostasis. This highlights the critical role of NATs in fine-tuning miRNA-mediated responses to abiotic stress [87].

**Table 1:** Key miRNA-regulated signaling pathways in plant stress tolerance.

Pathway	Key miRNAs	Target Genes	Stress Role	Refs.
Hormonal Pathways (ABA)	miR159, miR165/166, miR162, miR2105, miR5776-x, miR393	*ABI4*, *BG1*, *OsbZIP86*, *NCED3*, *PaNCED2*, *RACK1A*	Regulates ABA signaling, stomatal closure, root architecture, and dormancy	[48,49,50,51,52,53,54,55,56,57]
Hormonal Pathways (Auxin)	miR160, miR156ab, miR393	*ARF10*, *ARF16*, *ARF17*, *TIR1/AFB2*, *MdYUCCAs*, *MdPINs*, *MdLBD18*, *RACK1A*	Controls auxin signaling, root/shoot growth, and stress tolerance	[63,64,65,66,67,68,69]
Antioxidant Pathways	miR1119, csn-miR156f-2-5p, miR398	*SOD*, *CAT*, *CsSPL14*, *CSD1*, *CSD2*, *COX5b*	Maintains ROS homeostasis, photosynthetic efficiency, and antioxidant activity	[77,78,79]
Calcium Signaling Pathways	miR319, miR164, miR396	*TCP factors*, *NAC factors*, *GRFs*	Regulates calcium responses, water uptake, and stomatal behavior	[77,82,83,84]
NAT-Based Pathways	miR398 (NAT398b, NAT398c)	*ROS regulatory genes*	Optimizes miRNA responses, ROS regulation, and cellular balance	[87]

## 3. In Silico Tools for miRNA Target Prediction in Drought Tolerance Mechanisms

### 3.1. Overview of miRNA Target Prediction

MicroRNAs regulate gene expression by targeting specific mRNAs, which is a crucial step in understanding the mechanisms of drought tolerance in plants [88]. Investigating miRNA-mRNA interactions is essential for elucidating plant strategies in response to stress conditions and enhancing drought resistance [89]. To identify specific miRNA targets at the gene level, advanced molecular techniques such as Western blotting, microarrays, next-generation sequencing (NGS), and quantitative PCR are widely utilized [90,91]. However, these methods do not always enable precise determination of miRNA binding sites within the mRNA sequence. In this context, in silico tools, leveraging sequence complementarity and thermodynamic stability, provide a broad-scale prediction of potential miRNA-mRNA interactions, offering a cost-effective alternative to experimental approaches [92,93]. Such predictive methods facilitate the identification of stress-responsive genes involved in key processes, including ABA signaling, ROS detoxification, and root growth regulation [94,95,96]. Despite their effectiveness, in silico tools are constrained by false-positive results and incomplete genome annotations, particularly in non-model plants [97,98,99]. To address these limitations, combining in silico predictions with experimental validation methods enhances the reliability and accuracy of findings [100,101]. Moreover, advancements in artificial intelligence (AI) and machine learning models are unlocking new possibilities for exploring miRNA-regulated drought tolerance mechanisms. These technologies facilitate more precise predictions and contribute to a deeper understanding of plant stress response systems [102,103].

### 3.2. Applications of In Silico Tools for miRNA Research in Drought Tolerance

Predicting miRNA targets in plants is relatively easier compared to animals due to the extensive complementarity typically observed between plant miRNAs and their target RNAs, which simplifies computational analyses [104,105]. To achieve accurate target prediction in plants, advanced bioinformatics tools and web-based platforms such as psRNATarget, TargetFinder, miRTarBase, RNAhybrid, Tapir, and MirTarget have been developed (Table 2).

psRNATarget is a modern bioinformatics tool designed to predict the interaction of small RNAs (sRNAs), including miRNAs, with their target mRNAs in plants. This server identifies miRNA-mRNA interactions through complementary sequence analysis and assessment of target site accessibility [106]. Its updated version allows users to customize parameters, enabling the efficient identification of both canonical and non-canonical targets. psRNATarget, widely used as an in silico tool, has been employed to predict potential miRNA targets during drought conditions [107,108]. Its ability to process large datasets and work with transcript libraries makes it a key resource for studying miRNA mechanisms associated with drought tolerance in plants. Additionally, psRNATarget is extensively used in investigating major stress response pathways, such as ABA signaling and ROS detoxification [109,110].

One of the highly efficient and precise bioinformatics tools for predicting miRNA target genes in plants is TargetFinder. This tool employs a position-weighted scoring algorithm to evaluate the complementarity between miRNAs and their mRNA target regions [111]. The algorithm accounts for mismatches, gaps, and bulges, ensuring accurate and reliable predictions of miRNA-mRNA interactions [112].

miRTarBase is one of the largest and most reliable databases for experimentally validated miRNA-target interactions (MTIs), with over 3.8 million MTIs from around 13,690 studies [113]. It integrates diverse datasets, such as miRNA expression profiles, tissue-specific information, SNPs, and disease-associated variations, helping researchers understand miRNA regulation in different biological and environmental conditions, including drought stress [114,115]. In drought tolerance research, miRTarBase provides validated MTIs, enabling the identification of key regulatory pathways for stress responses, which is essential for studying plant adaptation [113,116]. By integrating expression data with validated targets, miRTarBase also aids in developing gene models and drought-tolerant plants [117].

RNAhybrid is a widely used in silico tool designed to predict interactions between miRNAs and mRNAs and identify the most energetically favorable binding regions. Its ability to accurately predict miRNA-mRNA duplexes makes RNAhybrid a crucial resource for studying the molecular mechanisms of plant adaptation to drought [118]. This tool facilitates the identification of key regulatory miRNAs, their functional validation, and their application in crop improvement programs [119]. The RNAhybrid tool is widely used for predicting interactions between miRNAs and plant mRNAs.

The next applications of in silico tools for miRNA target prediction include TAPIR, a web server designed to identify plant miRNA-mRNA interactions with both speed and precision. TAPIR can detect imperfectly matched duplexes that traditional tools often overlook. It employs two distinct algorithms: one based on the FASTA local alignment program for rapid genome-wide searches and another, more precise algorithm using RNAhybrid for detailed miRNA-mRNA interaction analysis [120]. The RNAhybrid-based algorithm is particularly advantageous for identifying complex regulatory phenomena such as “target mimicry,” where miRNA activity is sequestered. This feature makes TAPIR an invaluable resource for exploring regulatory networks under abiotic stresses, including drought. By accounting for parameters such as free energy and mismatches, TAPIR facilitates the discovery of miRNA-mediated pathways crucial for stress adaptation [121]. In the context of drought tolerance research, TAPIR enables researchers to investigate miRNA-mRNA interactions that regulate key stress responses, offering insights into both model and non-model plant species. This dual capability of speed and precision highlights TAPIR as a critical tool for advancing our understanding of miRNA roles in plant stress resilience [120,122].

MirTarget is a widely used bioinformatics tool for studying miRNAs in humans, animals, and plants. It identifies miRNA binding sites in mRNA sequences by evaluating free energy (ΔG) and other binding parameters. The program predicts miRNA binding sites in the 5′ untranslated region (5′UTR), coding sequence (CDS), and 3′ untranslated region (3′UTR) of mRNAs. MirTarget performs a detailed analysis of miRNA-mRNA interactions, including the starting points of binding sites, their locations within mRNA regions, pairing schemes, and the free energy (ΔG) of the interactions [123,124]. Additionally, the tool accounts for non-canonical pairings such as G–U and A–C, improving the precision of its predictions. These capabilities make MirTarget a comprehensive and versatile tool for investigating miRNA–target interactions across various organisms [125,126].

**Figure 3:**
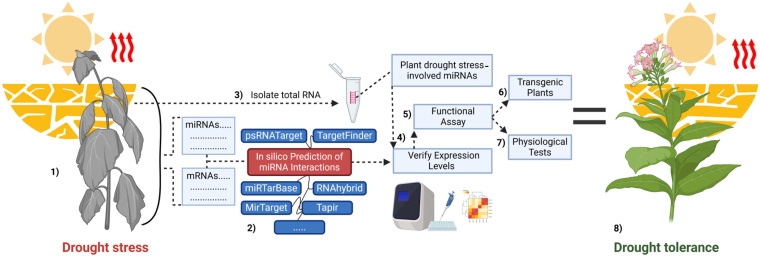
Integrated workflow for studying miRNA-mediated drought tolerance in plants. (1) Drought stress significantly impacts plants, causing physiological changes such as leaf yellowing, reduced photosynthesis, and excessive accumulation of ROS due to water deficiency. During this stress, drought-responsive miRNAs are activated. (2) Bioinformatics tools such as psRNATarget, TargetFinder, miRTarBase, RNAhybrid, and MirTarget are used to predict miRNA-mRNA interactions. These tools analyze miRNA binding sites on the mRNA sequence, providing insights into their regulatory mechanisms. (3) Total RNA is extracted from plant tissues (e.g., leaves and roots), serving as the foundation for studying miRNAs and their target mRNAs. (4) Methods like qPCR and Northern blot are employed to validate the in silico predictions of miRNAs and their target mRNAs. These techniques are used to determine inverse correlations in expression levels between miRNAs and their targets. (5) Functional analysis is performed to investigate whether miRNAs repress or activate mRNA expression. These studies reveal the specific genetic pathways regulated by miRNAs. (6) Transgenic plants are developed to overexpress or suppress specific miRNAs or their target genes. These models are then studied to evaluate their ability to adapt to drought stress. (7) Drought adaptation traits in transgenic plants, such as water retention capacity, root system architecture, and ROS levels, are assessed comprehensively. These analyses help determine the plants’ drought tolerance levels. (8) As a result of these steps, strategies to improve plant drought tolerance are identified. These strategies pave the way for developing drought-resistant crop varieties.

In the study of drought tolerance mechanisms, in silico tools are widely used to predict miRNA-mRNA interactions. These tools enable the identification of key stress response pathways, including ABA signaling, ROS detoxification, and root growth regulation. Additionally, the integration of RT-qPCR and degradome sequencing as experimental validation methods significantly enhances the reliability of the results (Figure 3). These approaches not only save time and are cost-effective but also help to define precise research directions [127]. In addition to widely used tools such as psRNATarget, TargetFinder, miRTarBase, RNAhybrid, Tapir, and MirTarget, modern tools like Cleaveland (for degradome analysis) [128] and DeepMirTar (for deep learning-based predictions) [129] further deepen the exploration of miRNA targets. These technologies, leveraging artificial intelligence and machine learning algorithms, contribute to a deeper understanding of the molecular regulatory mechanisms in plants under stress conditions and facilitate the development of drought-tolerant crop varieties.

The quantitative characteristics of miRNA-mRNA interactions (binding free energy and the ΔG/ΔGm ratio) cannot be directly determined through “wet” experiments but play a crucial role in understanding the competition between miRNAs for binding to mRNAs. For example, multiple miRNAs may compete to bind within the same mRNA cluster. In such cases, miRNAs with higher binding free energy will preferentially bind to the mRNA, enhancing their regulatory efficiency over the target gene [13,130]. These competitive mechanisms highlight the complex and dynamic role of miRNAs in regulating gene expression. Differences in binding free energy among various miRNAs determine the strength of their regulatory effects and their influence on specific target genes. Furthermore, understanding these interaction patterns offers significant insights into plant adaptive mechanisms under abiotic stresses, such as drought or salinity. Future research could focus on developing advanced bioinformatics tools to complement these quantitative characteristics with experimental methods. This would provide a deeper understanding of miRNA dynamics and inform strategies for improving plant resilience to stress conditions.

## 4. Experimental Approaches for miRNA Isolation and Analysis in Drought Stress Research

Isolating and analyzing miRNAs from plants is crucial for understanding their role in stress tolerance [131]. These methods help identify miRNAs, their target genes, and signaling pathways, as well as uncover mechanisms involved in the response to water scarcity, while refining and complementing bioinformatics predictions [132,133]. The development of RNA extraction kits, microarray analysis, high-throughput sequencing (HTS), and RACE-PCR methods has advanced this field significantly. These techniques provide precise, reliable, and scalable tools for discovering and validating miRNAs, while their bioinformatics integration opens the way for developing drought-resistant crops [134,135].

### 4.1. Isolation of Total RNA/miRNA

**Figure 4:**
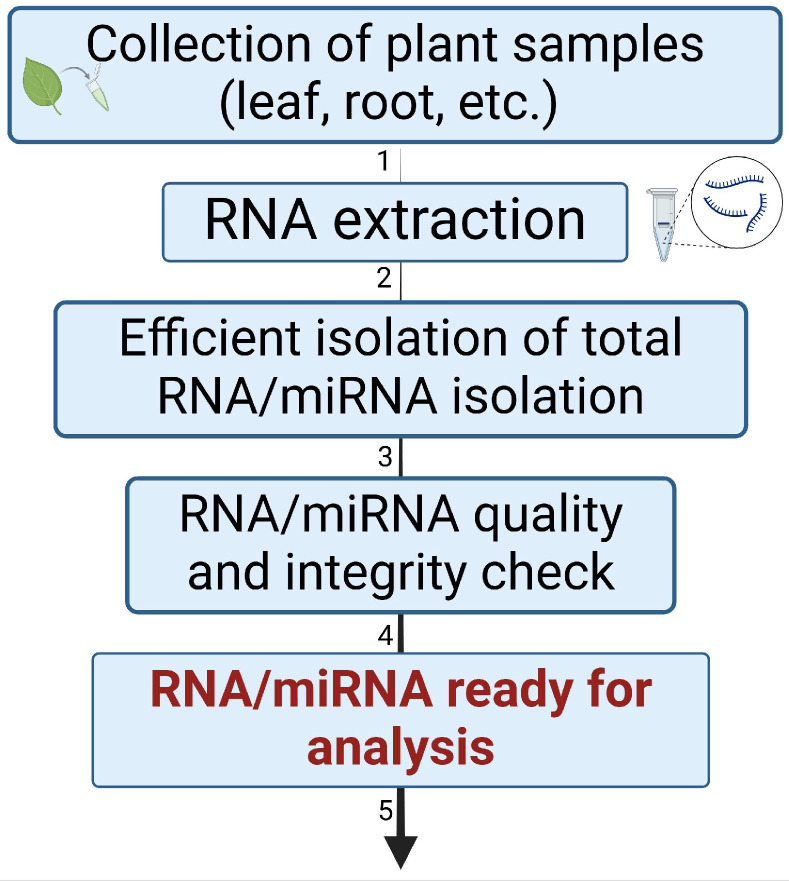
RNA extraction process for miRNA profiling and analysis. (1) Collection of plant samples subjected to drought stress (leaf, root, etc.). (2) RNA extraction using guanidine thiocyanate/phenol/chloroform extraction or commercial kits. (3) Ensuring efficient extraction while preserving small RNA fractions, including miRNA. (4) RNA/miRNA quality and integrity check using spectrophotometry, gel electrophoresis, or Bioanalyzer. (5) RNA/miRNA ready for analysis (miRNA profiling, HTS, or RACE-PCR).

High-quality RNA extraction is fundamental to miRNA research, as it ensures the accuracy of miRNA expression profiling, HTS, and RACE-PCR analyses. MiRNAs, being smaller and less concentrated than total RNA, require specialized methods for efficient isolation [136,137]. Techniques such as guanidine thiocyanate/phenol/chloroform extraction, Trizol, or other commercially available kits are used. Modern extraction kits, such as Qiagen’s miRNeasy and Invitrogen’s PureLink, efficiently isolate total RNA while preserving small RNA fractions. RNA should always be isolated from fresh tissues or tissues stored frozen, as RNA has a short half-life [138,139]. Extracting RNA from plant tissues can be challenging due to the presence of polysaccharides and phenolic compounds. After RNA extraction, its quality and integrity must be checked using methods such as spectrophotometric analysis, gel electrophoresis, or tools like Bioanalyzer or TapeStation (Figure 4) [140,141].

### 4.2. Microarray Analysis for miRNA Profiling

Microarray analysis is a high-throughput method that allows for the simultaneous measurement of miRNA expression levels under stress conditions, such as drought [142]. This technique provides crucial information for understanding the role of miRNAs in plant stress responses. Microarray analysis is used to identify differentially expressed miRNAs under various stress conditions, such as drought, salinity, and temperature [143]. For example, in an *A. thaliana* study, several miRNAs, including miR164, miR169, miR393, and miR396, were found to be involved in drought tolerance and pathogen resistance responses [144,145]. In a *M. truncatula* study, miR169 and miR396 were highly expressed during drought, while miR398 played a key role in oxidative stress tolerance. Microarray analysis not only identifies stress-responsive miRNAs but also helps in identifying the regulatory networks involving their target genes [146,147]. This method helps to understand the mechanisms of miRNA responses to stress and, by integrating with other methods such as HTS, enables the discovery of novel miRNAs, providing comprehensive information about miRNA expression and function.

### 4.3. High-Throughput Sequencing for miRNA Discovery

High-throughput sequencing (HTS) has introduced a significant breakthrough in the field of miRNA discovery and characterization, as this method allows for comprehensive and detailed profiling of miRNA expression [148]. Unlike microarray analysis, HTS is not limited to known sequences, enabling the identification of novel miRNA species. This method is an essential tool for studying the full spectrum of miRNA sequences, particularly in plants subjected to environmental stresses such as drought, salinity, and temperature [149,150]. HTS methods, including RNA-seq, sRNA-seq, and RNA-PET-seq platforms, have significantly advanced miRNA research in plants. These technologies provide a more comprehensive and efficient identification of miRNAs compared to traditional methods, as they offer high coverage and depth for genomic and transcriptomic studies. HTS platforms allow for the identification of novel miRNA species and their interactions with target genes through millions of short sequencing reads. For example, the sRNA-seq method enables the study of the spatial and temporal accumulation of miRNAs in plant tissues, which is particularly important for analyzing mature miRNAs that are more stable than precursor miRNA fragments [151,152,153]. Additionally, HTS methods like RNA-PET-seq enable the precise mapping of the transcriptional regions of miRNA genes, helping to identify new miRNA species and their target genes. Combining HTS data with other methods, such as degradome-seq and dsRNA-seq, enhances the accuracy of miRNA annotations and allows for the study of miRNA biogenesis and processing. Thus, HTS is an essential tool for miRNA discovery and functional analysis, especially in plants subjected to environmental stresses like drought [154,155]. Recent studies, using HTS, have identified miRNAs in plants that respond to drought. For example, in *Camellia oleifera*, it was shown that miR398, miR408-3p, miR166, and several novel miRNAs regulate drought tolerance. These studies provide insights into how regulatory networks mediated by miRNAs contribute to enhancing drought resistance [156]. Similarly, in tomato plants, HTS has identified several key miRNAs involved in drought tolerance under drought stress. Specifically, conserved miRNAs such as sly-miR156a, sly-miR164a-5p, sly-miR171c, and sly-miR396a-3p were found to be differentially expressed under drought conditions, with some showing higher expression in the drought-tolerant IL9–1 line and lower expression in the drought-sensitive M82 line. Additionally, novel miRNAs such as sly_miN_702, sly_miN_709, and sly_miN_710 were also identified as differentially expressed in response to drought stress, indicating their potential role in drought tolerance. These miRNAs regulate a variety of stress-responsive genes, including transcription factors and protein kinases [157]. Such findings demonstrate the effectiveness of the HTS method in uncovering the complex regulatory networks mediated by miRNAs that contribute to enhancing drought tolerance in plants.

### 4.4. RACE-PCR for miRNA Target Validation

Rapid amplification of cDNA ends–PCR (RACE-PCR) is widely used for validating miRNA target genes, enabling confirmation of the regulatory relationship between miRNAs and their specific target genes. This method precisely identifies the 3′ or 5′ UTR regions of the target mRNA [158,159]. In a study of *A. thaliana*, new miRNAs responsive to ABA were identified, including ath-miRn-1, ath-miRn-2, and others, with their potential target genes predicted using the psRNATarget bioinformatics tool. Validation using the 5′ RLM-RACE-PCR method revealed that the target genes of these miRNAs, such as AT1G73390.3 and AT5G40550.1, were identified as specific sites of mRNA degradation. These genes are involved in cellular processes, including endosomal targeting and anthocyanin pigment production, indicating the regulatory role of these miRNAs in ABA signaling. The 5′ RLM-RACE results provide strong evidence that these miRNAs regulate gene expression post-transcriptionally in *Arabidopsis*, confirming their role in plant stress responses [160]. Moreover, the RACE-PCR method was used to validate miRNA target genes under drought stress conditions. For example, in a study of *O. sativa*, several drought-responsive miRNAs, including miR156b-3p, miR159a.2, miR164c, miR169d, miR172d-5p, miR396g, miR395a, miR528-3p, and miR812q, along with their target genes, were identified. Through the use of RACE-PCR, this research contributed to understanding the molecular mechanisms of drought tolerance in rice and confirmed the interactions of these miRNAs with stress-responsive genes [161]. Additionally, a study by Fu et al. [162] investigated the role of miR159a in response to drought stress in Populus euphratica. Using the RACE-PCR method, the researchers validated the target genes of miR159a and confirmed its positive regulatory role in drought response. In another study, Gossypium hirsutum was investigated for miRNAs involved in the plant’s response to salt and drought stresses. For example, the study identified miRNVL5, which regulates the GhCHR gene, encoding a zinc-finger protein. RACE-PCR was used to validate the interaction between miRNVL5 and GhCHR, contributing to the understanding of stress tolerance mechanisms in cotton [163]. These discoveries also pave the way for future biotechnological applications, such as the development of genetically modified crops with enhanced resistance to drought and other abiotic stresses.

## 5. Conclusions and Prospects

In conclusion, miRNAs are key regulators of gene expression and play a crucial role in plant adaptation to drought stress. By regulating various signaling pathways, such as ABA signaling, ROS detoxification, and root system optimization, miRNAs significantly enhance plant tolerance to water scarcity. This review highlights the latest advancements in understanding the mechanisms of drought-responsive miRNAs, their target genes, and associated regulatory networks. In the future, integrating bioinformatics tools such as psRNATarget, TargetFinder, and MirTarget with experimental methods will provide effective strategies for identifying and validating miRNA-mRNA interactions. These integrative approaches will deepen our understanding of miRNA-mediated stress responses and open new opportunities for developing drought-resistant crops. Furthermore, experimental techniques such as miRNA isolation, microarray analysis, HTS, and RACE-PCR contribute significantly to understanding the mechanisms of drought tolerance mediated by miRNAs. These methods complement bioinformatics predictions and provide tools for the precise identification of miRNAs in response to ecological stress factors. Additionally, genomic editing technologies such as CRISPR/Cas9 offer unique opportunities to precisely regulate miRNA expression and their target pathways, improving plant adaptation to abiotic stresses. Future research should focus on expanding miRNA studies in agriculturally significant, less-studied plants. The use of advanced technologies, such as single-cell sequencing and artificial intelligence, to predict miRNA targets accurately will further enhance our understanding. These efforts will make significant contributions to the development of drought-resistant crop varieties and strengthen global food security in the face of environmental challenges.

## Figures and Tables

**Table 2:** In silico tools for miRNA-mRNA interaction prediction in drought tolerance studies.

Tool	Description	miRNAs Studied for Drought Tolerance	Refs.
psRNATarget	Predicts miRNA-mRNA interactions via sequence complementarity and target accessibility. Used for studying drought pathways like ABA signaling and ROS detoxification	miR159a, miR1119, miR156d-3p, miR160a-5p, miR162a-3p, miR172b-3p, miR398a-5p, Novel_8, Novel_9, Novel_105	[106,107,108,109,110]
TargetFinder	Uses a position-weighted scoring algorithm to evaluate miRNA-mRNA complementarity	miR171, miR319, miR398, miR1432, miR156, miR396	[111,112]
miRTarBase	A database of experimentally validated miRNA–target interactions	miRNAs involved in ABA signaling and stress response pathways	[113,114,115,116,117]
RNAhybrid	Predicts energetically favorable miRNA-mRNA duplexes	miR160, miR164, miR166, miR393, miR529, miR169, miR2275	[118,119]
Tapir	Predicts imperfectly matched miRNA-mRNA interactions using FASTA- and RNAhybrid-based algorithms	miR172, miR164, miR160	[120,121,122]
MirTarget	Analyzes miRNA binding sites in coding and untranslated regions of mRNAs	tae-miR1127b-3p, tae-miR159a,b-3p, tae-miR164-5p, tae-miR171a-3p	[123,124,125,126]

## Data Availability

The data supporting this study are available from the corresponding author upon reasonable request.
